# Association Between Temporary Employment and Current Smoking and Change in Smoking Behaviors: A Prospective Cohort Study From South Korea (2009–2018)

**DOI:** 10.2188/jea.JE20230223

**Published:** 2024-10-05

**Authors:** Seong-Uk Baek, Min-Seok Kim, Myeong-Hun Lim, Taeyeon Kim, Jin-Ha Yoon, Yu-Min Lee, Jong-Uk Won

**Affiliations:** 1 Department of Occupational and Environmental Medicine, Severance Hospital, Yonsei University College of Medicine, Seoul, Korea; 2 The Institute for Occupational Health, Yonsei University College of Medicine, Seoul, Korea; 3 Graduate School, Yonsei University College of Medicine, Seoul, Korea; 4 Department of Public Health, Graduate School of Yonsei University, Seoul, Korea; 5 Department of Preventive Medicine, Yonsei University College of Medicine, Seoul, Korea

**Keywords:** tobacco, cigarette smoking, precarious employment, smoking initiation, smoking cessation, repeated measures analysis

## Abstract

**Background:**

Previous studies have suggested that employment insecurity is associated with adverse health outcomes. We explored the association between temporary employment and smoking behaviors.

**Methods:**

We analyzed 11,795 workers (51,867 observations) from the Korea Health Panel Study (2009–2018). Employment types were categorized as regular, fixed-term, or daily, based on the duration of labor contract. The outcomes were current smoking status and changes in smoking behavior (initiation or cessation) in the following year. Generalized estimating equations were used to estimate odds ratios (ORs) and 95% confidence intervals (CIs).

**Results:**

The proportions of fixed-term and daily workers were 41.2% and 16.4% for women and 23.6% and 12.4% for men, respectively. Temporary employment was associated with increased odds of current smoking, while also demonstrating prospective associations with changes in smoking behaviors. For instance, in prospective analyses, male workers with fixed-term and daily employments were associated with a decreased likelihood of smoking cessation (OR 0.77; 95% CI, 0.65–0.91 for fixed-term employment and OR 0.66; 95% CI, 0.52–0.83 for daily employment) in the following year compared to those with regular employment. Moreover, those experiencing consecutive temporary employment was most inversely associated with smoking cessation in both men (OR 0.56; 95% CI, 0.44–0.71) and women (OR 0.37; 95% CI, 0.16–0.85) compared to those experiencing consecutive regular employment. However, no clear association between temporary employment and smoking initiation was observed in both men and women.

**Conclusion:**

Temporary employment is directly associated with current smoking and inversely associated with smoking cessation. Policies are needed to improve job insecurity among temporary employees.

## INTRODUCTION

Due to rapid changes in the structure of the labor market, nonstandard forms of employment arrangements have increased globally in the last few years,^1^^–^^3^ raising substantial public health concerns due to their potential impact on health.^4^ This trend has been accelerated by the recent coronavirus disease 2019 pandemic, which has caused more workers to engage in short-term task-based work arrangements.^5^

Temporary employment refers to work arrangements that last for a finite period, often ranging from a few weeks to a few years, depending on the industry, company, or job function. In some cases, temporary employment may be project-based, seasonal, or designed to fulfill temporary staffing requirements. Temporary employment arrangements is a major element of precarious work, posing workers with high job insecurity, unpredictable work schedules, and lack of legal protection or employment benefits.^6^ Previous studies found that temporary employment is associated with various negative health outcomes. For example, a meta-analysis conducted by Virtanen et al demonstrated that temporary workers exhibit a higher risk of psychological distress, musculoskeletal disorders, and mortality.^7^ Other recent studies found that workers with temporary employment experience increased mortality from various chronic or acute diseases, including cardiovascular and respiratory diseases, cancer, injury, and suicide.^8^^,^^9^ Along with fatal outcomes, temporary employment is directly associated with mental health problems, such as depressive symptoms and poor psychological well-being.^10^^–^^13^

Given that smoking is a known risk factor for a variety of diseases, such as ischemic heart disease and cancer, the increased prevalence of smoking among temporary employees may contribute to a higher mortality risk in this population. However, in the current literature, only a limited number of studies have investigated how temporary employment is associated with workers’ smoking habits, and previous studies have produced mixed findings. For instance, a previous longitudinal analysis conducted by Jung et al have found that temporary employment is associated with an increased risk of smoking in male workers.^14^ However, other cross-sectional studies have found that this association is observed only in women^15^^,^^16^ or even found no significant relationship between temporary employment and smoking behavior.^17^ The inconsistencies in the existing literature can be attributable to several factors. First, individual studies have included relatively small sample sizes or observations that were limited to specific occupations. Second, previous studies have not distinguished between different types of temporary employment contracts, such as fixed-term employment and daily employment. For instance, temporary employees who work for several months in the same workplace are exposed to different employment conditions than daily workers who work under task-based arrangements. Furthermore, within the current literature, most studies have examined the cross-sectional association between temporary employment and current smoking, leaving a research gap in understanding how temporary employment can influence change in smoking behaviors over time.

Therefore, our study aimed to investigate (i) the association of temporary employment with current smoking, high-intensity smoking, and initiation and cessation of smoking, and (ii) how changes in employment position and persistent temporary employment are associated with changes in smoking behavior. By examining the relationship between temporary employment and smoking behaviors, our research will provide insights into the mechanisms through which temporary employment may contribute to the development of negative health outcomes.

## METHODS

### Study sample

The study sample was drawn from the Korea Health Panel Study (KHPS), which has been conducted annually by the National Health Insurance Service and the Korea Institute for Health and Social Affairs since 2008. To systemically select survey participants that were representative of the population in South Korea, the KHPS employed two-stage stratified cluster sampling in which enumeration districts in South Korea were regarded as primary sampling units, and households in each enumeration district were selected as secondary sampling units. Trained interviewers conducted face-to-face, one-on-one surveys of all selected households. In 2008, the initial survey year, 24,616 survey participants from 7,866 households were included and followed up annually thereafter. The sample participation rate dropped by approximately 2–5% each year. In the last survey year, 2018, 17,550 participants were surveyed.

Survey participants from 2009 to 2018 were included because the survey questionnaire on smoking behavior was introduced in 2009. First, we included 21,913 participants aged >18 years who participated in at least one wave from 2009 to 2018. We limited our sample to employed workers, leaving 12,049 participants and 53,585 observations available. Finally, participants with missing values were excluded, yielding 11,795 participants and 51,867 observations (eTable 1).

### Data availability and ethics statement

Raw KHPS data are available upon request from the Korea Institute for Health and Social Affairs (https://www.khp.re.kr:444/eng/data/data.do). The Institutional Review Board of author’s institution reviewed and approved the present study (4–2023–0145). Written informed consent was obtained from all participants.

### Variables

#### Employment type

The employment type of workers was repeatedly measured by survey year. Based on the classification of the Korean National Statistical Office (KSNO), we categorized employment types into three groups: “regular,” “fixed-term,” and “daily employment.” According to the KNSO, regular employment refers to employment contracts that do not have a finite period or a period of 1 year or more. Fixed-term employment included workers with contracts lasting 1 month or longer but less than 1 year, while daily employment included workers with contracts lasting less than 1 month. The KSNO classifies permanent employment and long-term temporary employment (with a contract duration of 1 year or more) into a single category as regular employment because any labor contract lasting more than 1 year is subject to the same retirement pay, bonuses, and social benefits under Korean labor law as permanent employment.^18^ This classification has been used in previous studies on the health effect of temporary employment.^19^^–^^21^

#### Smoking behaviors

The smoking status of survey participants was repeatedly measured by survey year. Survey participants were asked, “Are you currently smoking?” If yes, they were classified as current smokers and asked, “How many cigarettes do you smoke a day on average?” Those who smoked ≥20 cigarettes a day on average were classified as high-intensity smokers. Next, we assessed changes in smoking behavior by comparing the smoking status of each year of concern with that of the following year. Specifically, for each year*_t_*, those who reported that they did not smoke in the concerned year (year*_t_*) but did so in the following year (year*_t_*_+1_) were classified as having initiated smoking. Conversely, those who reported they smoked in the concerned year (year*_t_*) but did not in the following year (year*_t_*_+1_) were classified as having ceased smoking.

#### Covariates

We adjusted for demographic, socioeconomic, and occupational characteristics in the regression analysis as time-varying covariates. Gender (“Men”, “Women”) was adjusted. Age was categorized as “<30 years,” “30–39 years,” “40–49 years,” “50–59 years,” and “≥60 years.” Education level was categorized as having completed “middle school or below,” “high school,” and “college or above.” Income level was categorized into three groups (Q1–Q3) according to the tertile values of the total household income for each year. Marital status was categorized as “married,” “unmarried,” and “others (divorced, separated, or widowed).” Occupation type was categorized as “blue collar,” “service or sales workers,” and “white collar” based on the Korean Standard Classification of Occupation, seventh version. We also included binary dummy variables representing each survey year in the regression models to control for wave-specific effects.

### Statistical analysis

#### Descriptive analysis

First, we compared the features of the study sample according to employment type (regular, fixed-term, and daily employments). Next, we calculated the smoking prevalence according to each study variable. For descriptive analyses, chi-square test was used to compare the characteristics.

#### Regression analysis

First, we explored the cross-sectional association between temporary employment and the odds of current and high-intensity smoking. Specifically, for each survey year*_t_*, we examined whether temporary employment in year*_t_* is associated with current smoking or high-intensity smoking in year*_t_*. Subsequently, we explored the prospective association between temporary employment and initiation and cessation of smoking. For smoking initiation and cessation analyses, we only included those with information about the smoking status in the following year. Specifically, for each survey year*_t_*, we examined whether temporary employment in year*_t_* is prospectively associated with initiation or cessation of smoking in year*_t_*_+1_ while controlling for the confounding effect of covariates in year*_t_*.

Finally, we explored how changes in employment position and persistent temporary employment were associated with changes in smoking behaviors. Specifically, according to the employment positions of each concerned year (year*_t_*) and the following year (year*_t_*_+1_), we categorized the changes in employment status into “Regular → Regular,” “Temporary (either fixed-term or daily employment) → Regular,” “Regular → Temporary,” and “Temporary → Temporary.” In this analysis, we examined how change in employment status (year*_t_* → year*_t_*_+1_) was associated with change in smoking behavior (year*_t_* → year*_t_*_+1_) while controlling the confounding effect of covariates in year*_t_*. By doing so, we investigated how change in employment position or consecutive exposure to temporary employment was associated with initiation and cessation of smoking.

For all regression analyses, we employed generalized estimating equation (GEE) models to perform repeated measurements for each participant. Based on the value of the quasi-likelihood information criterion, we selected an independent working correlation matrix for GEE analyses.^22^ Effect sizes were presented as odds ratios (ORs) and 95% confidence intervals (CIs). R software (version 4.2.3; R Foundation for Statistical Computing, Vienna, Austria) was used for all statistical analyses and visualizations. The R package ‘*geepack*’ and function ‘*geeglm*’ were used to fit the GEE models.

## RESULTS

### Descriptive analysis

Table 1 shows the characteristics of the observations according to employment type. The overall sample comprised 29,131 men (56.2%) and 22,736 women (43.8%). The observations comprised 54.5% regular employment, 31.3% fixed-term employment, and 14.1% daily employment. The proportion of fixed-term employment (women: 41.2% vs men: 23.6%) and daily employment (women: 16.4% vs men: 12.4%) was higher among women than among men. For both sexes, the proportions of older workers, low-education level, low-income level, and blue-collar workers were the highest among workers with daily employment, followed by those with fixed-term and regular employments.

**Table 1.** Characteristics of study samples according to employment types throughout the whole observational period

Characteristics	All				Men				Women			

	Employment type			*P* value^*^	Employment type			*P* value^*^	Employment type			*P* value^*^
		
	Regular (*N* = 28,283)	Fixed-term (*N* = 16,251)	Daily (*N* = 7,333)		Regular (*N* = 18,648)	Fixed-term (*N* = 6,884)	Daily (*N* = 3,599)		Regular (*N* = 9,635)	Fixed-term (*N* = 9,367)	Daily (*N* = 3,734)	
Age, years												
<30	3,549 (12.5)	2,220 (13.7)	651 (8.9)	<0.001	1,428 (7.7)	947 (13.8)	318 (8.8)	<0.001	2,121 (22.0)	1,273 (13.6)	333 (8.9)	<0.001
30–39	8,232 (29.1)	3,018 (18.6)	598 (8.2)		5,454 (29.2)	1,389 (20.2)	291 (8.1)		2,778 (28.8)	1,629 (17.4)	307 (8.2)	
40–49	9,419 (33.3)	4,210 (25.9)	1,614 (22.0)		6,653 (35.7)	1,454 (21.1)	827 (23.0)		2,766 (28.7)	2,756 (29.4)	787 (21.1)	
50–59	5,425 (19.2)	3,283 (20.2)	2,089 (28.5)		3,849 (20.6)	1,150 (16.7)	1,121 (31.1)		1,576 (16.4)	2,133 (22.8)	968 (25.9)	
≥60	1,658 (5.9)	3,520 (21.7)	2,381 (32.5)		1,264 (6.8)	1,944 (28.2)	1,042 (29.0)		394 (4.1)	1,576 (16.8)	1,339 (35.9)	
Education												
Middle school ​ or below	1,759 (6.2)	3,583 (22.0)	3,274 (44.6)	<0.001	975 (5.2)	1,216 (17.7)	1,398 (38.8)	<0.001	784 (8.1)	2,367 (25.3)	1,876 (50.2)	<0.001
High school	9,447 (33.4)	7,254 (44.6)	3,282 (44.8)		6,298 (33.8)	3,259 (47.3)	1,825 (50.7)		3,149 (32.7)	3,995 (42.6)	1,457 (39.0)	
College or above	17,077 (60.4)	5,414 (33.3)	777 (10.6)		11,375 (61.0)	2,409 (35.0)	376 (10.4)		5,702 (59.2)	3,005 (32.1)	401 (10.7)	
Income												
Q1	5,941 (21.0)	6,849 (42.1)	4,618 (63.0)	<0.001	3,899 (20.9)	3,229 (46.9)	2,328 (64.7)	<0.001	2,042 (21.2)	3,620 (38.6)	2,290 (61.3)	<0.001
Q2	10,003 (35.4)	5,497 (33.8)	1,878 (25.6)		6,890 (36.9)	2,279 (33.1)	924 (25.7)		3,113 (32.3)	3,218 (34.4)	954 (25.5)	
Q3	12,339 (43.6)	3,905 (24.0)	837 (11.4)		7,859 (42.1)	1,376 (20.0)	347 (9.6)		4,480 (46.5)	2,529 (27.0)	490 (13.1)	
Marital status												
Married	20,615 (72.9)	10,881 (67.0)	4,919 (67.1)	<0.001	14,735 (79.0)	4,595 (66.7)	2,514 (69.9)	<0.001	5,880 (61.0)	6,286 (67.1)	2,405 (64.4)	<0.001
Unmarried	6,585 (23.3)	3,683 (22.7)	1,110 (15.1)		3,465 (18.6)	1,910 (27.7)	736 (20.5)		3,120 (32.4)	1,773 (18.9)	374 (10.0)	
Others	1,083 (3.8)	1,687 (10.4)	1,304 (17.8)		448 (2.4)	379 (5.5)	349 (9.7)		635 (6.6)	1,308 (14.0)	955 (25.6)	
Occupation												
Blue collar	8,911 (31.5)	7,252 (44.6)	5,524 (75.3)	<0.001	7,221 (38.7)	4,308 (62.6)	3,297 (91.6)	<0.001	1,690 (17.5)	2,944 (31.4)	2,227 (59.6)	<0.001
Service and ​ sale worker	3,266 (11.5)	4,343 (26.7)	1,473 (20.1)		1,838 (9.9)	1,113 (16.2)	212 (5.9)		1,428 (14.8)	3,230 (34.5)	1,261 (33.8)	
White collar	16,106 (56.9)	4,656 (28.7)	336 (4.6)		9,589 (51.4)	1,463 (21.3)	90 (2.5)		6,517 (67.6)	3,193 (34.1)	246 (6.6)	

^*^Chi-square test.

Table 2 shows smoking prevalence according to sample characteristics. For both men and women, smoking prevalence was the highest among workers with daily employment (men: 57.3% and women: 4.6%), followed by fixed-term employment (men: 46.5% and women: 2.7%) and regular employment (men: 44.7% and women: 1.5%). Additionally, smoking prevalence was high among workers with low-income levels, other types of marital status, and blue-collar jobs for both sexes.

**Table 2.** Smoking prevalence according to study variables throughout the whole observational period

Characteristics	All			Men			Women		

	Smoking status		*P* value^*^	Smoking status		*P* value^*^	Smoking status		*P* value^*^
		
	Current smoker *N* = 14,157	Non-smoker *N* = 37,710		Current smoker *N* = 13,587	Non-smoker *N* = 15,544		Current smoker *N* = 570	Non-smoker *N* = 22,166	
Employment type									
Regular	8,481 (30.0)	19,802 (70.0)	<0.001	8,337 (44.7)	10,311 (55.3)	<0.001	144 (1.5)	9,491 (98.5)	<0.001
Fixed-term	3,440 (21.2)	12,811 (78.8)		3,187 (46.3)	3,697 (53.7)		253 (2.7)	9,114 (97.3)	
Daily	2,236 (30.5)	5,097 (69.5)		2,063 (57.3)	1,536 (42.7)		173 (4.6)	3,561 (95.4)	
Age, years									
<30	1,377 (21.4)	5,043 (78.6)	<0.001	1,283 (47.6)	1,410 (52.4)	<0.001	94 (2.5)	3,633 (97.5)	0.132
30–39	3,962 (33.4)	7,886 (66.6)		3,865 (54.2)	3,269 (45.8)		97 (2.1)	4,617 (97.9)	
40–49	4,722 (31.0)	10,521 (69.0)		4,552 (51.0)	4,382 (49.0)		170 (2.7)	6,139 (97.3)	
50–59	2,703 (25.0)	8,094 (75.0)		2,590 (42.3)	3,530 (57.7)		113 (2.4)	4,564 (97.6)	
≥60	1,393 (18.4)	6,166 (81.6)		1,297 (30.5)	2,953 (69.5)		96 (2.9)	3,213 (97.1)	
Education									
Middle school or below	1,736 (20.1)	6,880 (79.9)	<0.001	1,557 (43.4)	2,032 (56.6)	<0.001	179 (3.6)	4,848 (96.4)	<0.001
High school	6,282 (31.4)	13,701 (68.6)		5,999 (52.7)	5,383 (47.3)		283 (3.3)	8,318 (96.7)	
College or above	6,139 (26.4)	17,129 (73.6)		6,031 (42.6)	8,129 (57.4)		108 (1.2)	9,000 (98.8)	
Income									
Q1	5,078 (29.2)	12,330 (70.8)	<0.001	4,725 (50.0)	4,731 (50.0)	<0.001	353 (4.4)	7,599 (95.6)	<0.001
Q2	4,904 (28.2)	12,474 (71.8)		4,770 (47.3)	5,323 (52.7)		134 (1.8)	7,151 (98.2)	
Q3	4,175 (24.4)	12,906 (75.6)		4,092 (42.7)	5,490 (57.3)		83 (1.1)	7,416 (98.9)	
Marital status									
Married	9,818 (27.0)	26,597 (73.0)	<0.001	9,582 (43.9)	12,262 (56.1)	<0.001	236 (1.6)	14,335 (98.4)	<0.001
Unmarried	3,438 (30.2)	7,940 (69.8)		3,295 (53.9)	2,816 (46.1)		143 (2.7)	5,124 (97.3)	
Others	901 (22.1)	3,173 (77.9)		710 (60.4)	466 (39.6)		191 (6.6)	2,707 (93.4)	
Occupation									
Blue collar	7,629 (35.2)	14,058 (64.8)	<0.001	7,394 (49.9)	7,432 (50.1)	<0.001	235 (3.4)	6,626 (96.6)	<0.001
Service and sale worker	1,884 (20.7)	7,198 (79.3)		1,681 (53.1)	1,482 (46.9)		203 (3.4)	5,716 (96.6)	
White collar	4,644 (22.0)	16,454 (78.0)		4,512 (40.5)	6,630 (59.5)		132 (1.3)	9,824 (98.7)	

^*^Chi-square test.

eFigure 1 presents annual trends in the prevalence of smoking and temporary employment. While smoking prevalence and the proportion of daily employment showed a steady decreasing trend, the proportion of fixed-term employment temporarily increased in mid-2010.

### Cross-sectional analysis

Table 3 presents the association between temporary employment and smoking-related outcomes. In the cross-sectional analysis, fixed-term employment was significantly associated with a higher OR of current smoking (OR 1.12; 95% CI, 1.02–1.23), while daily employment was associated with an even higher OR (OR 1.72; 95% CI, 1.49–2.00) compared to regular employment after controlling for confounding variables. Additionally, both fixed-term employment (OR 1.36; 95% CI, 1.21–1.52) and daily employment (OR 2.10; 95% CI, 1.79–2.46) was associated with high-intensity smoking compared to regular employment.

**Table 3.** Association between temporary employment and smoking behaviors

	Current smoking			High-intensity smoking		

	Cases/*N*	Model A	Model B	Cases/*N*	Model A	Model B
	
		OR (95% CI)	OR (95% CI)		OR (95% CI)	OR (95% CI)
**Employment type**						
Regular	8,481/28,283	Reference	Reference	3,046/28,283	Reference	Reference
Fixed-term	3,440/16,251	0.63 (0.58–0.68)	1.12 (1.02–1.23)	1,478/16,251	0.83 (0.75–0.92)	1.36 (1.21–1.52)
Daily	2,236/7,333	1.02 (0.92–1.14)	1.72 (1.49–2.00)	1,234/7,333	1.68 (1.47–1.91)	2.10 (1.79–2.46)

	Smoking initiation			Smoking cessation		

	Cases/*N*	Model A	Model B	Cases/*N*	Model A	Model B
	
		OR (95% CI)	OR (95% CI)		OR (95% CI)	OR (95% CI)

**Employment type**						
Regular	683/15,836	Reference	Reference	854/6,965	Reference	Reference
Fixed-term	258/10,804	0.80 (0.71–0.89)	0.93 (0.78–1.10)	317/2,932	0.87 (0.75–1.01)	0.80 (0.69–0.94)
Daily	108/4,299	0.88 (0.75–1.02)	1.03 (0.80–1.33)	167/1,923	0.69 (0.58–0.83)	0.68 (0.54–0.84)

CI, confidence interval; OR, odds ratio.

Model A: Unadjusted model.

Model B: Gender, age, education, income, marital status, occupation, and survey year were adjusted.

**Figure 1.**
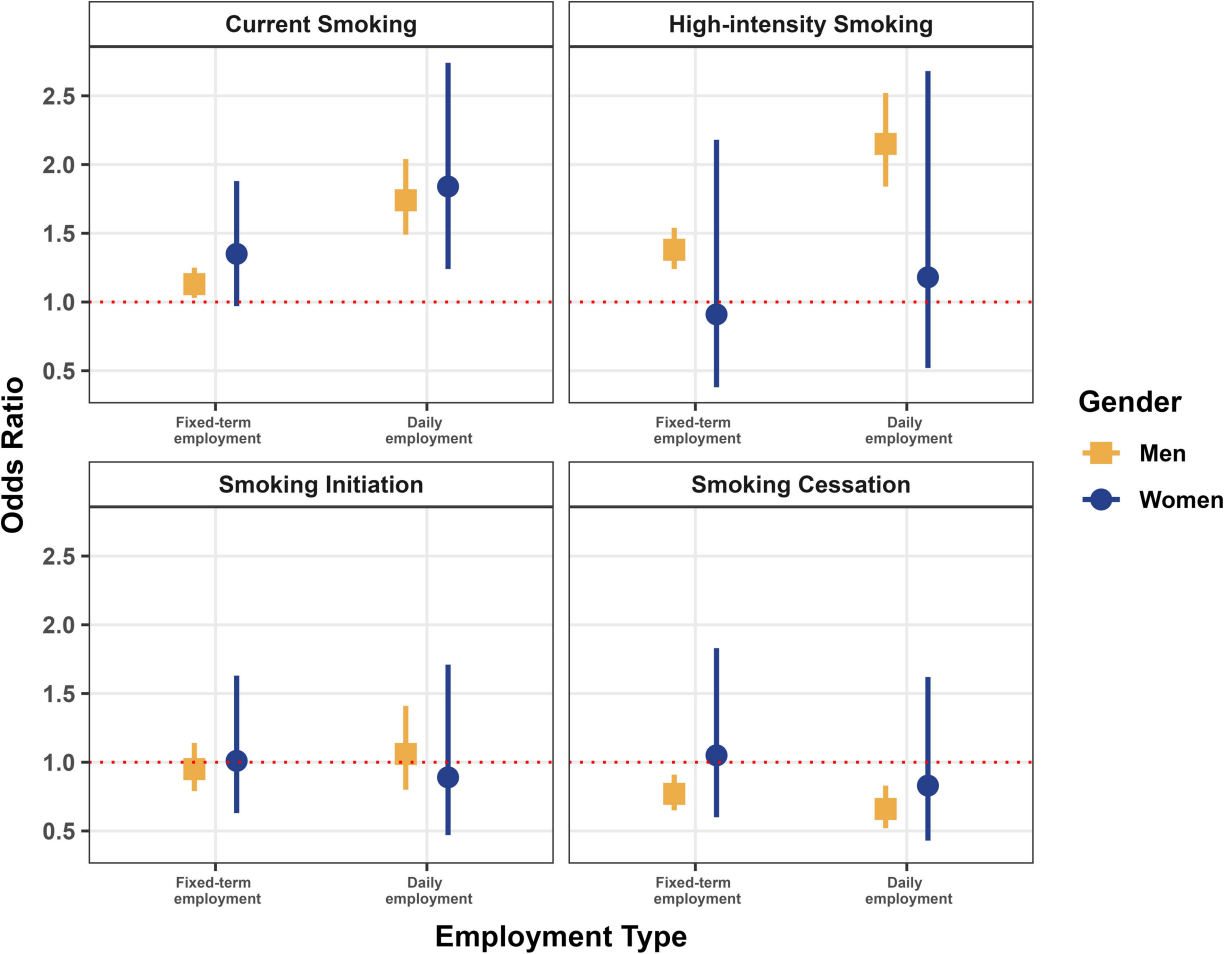
Association between temporary employment and smoking behaviors in men and women. The models adjusted for gender, age, education, income, marital status, occupation, and survey year (reference: regular employment).

Figure 1 and eTable 2 shows the results of gender-stratified analyses. For male workers, fixed-term and daily employments were associated with an increased OR of current smoking (OR 1.13; 95% CI, 1.03–1.25 for fixed-term employment and OR 1.74; 95% CI, 1.49–2.04 for daily employment). For female workers, daily employment was associated with an increased OR of current smoking (OR 1.84; 95% CI, 1.24–2.74).

### Prospective analysis

The results from Table 3 shows that those with fixed-term employment (OR 0.80; 95% CI, 0.69–0.94) and daily employment (OR 0.68; 95% CI, 0.54–0.84) were less likely to quit smoking in the following year compared to those with regular employment. However, no clear associations between temporary employment and smoking initiation in the following year.

Figure 1 and eTable 2 shows that for male workers, fixed-term and daily employments were associated with a decreased likelihood of smoking cessation (OR 0.77; 95% CI, 0.65–0.91 for fixed-term employment and OR 0.66; 95% CI, 0.52–0.83 for daily employment). However, no clear association was observed between temporary employment and change in smoking behavior in female workers.

Table 4 shows how changes in employment types were associated with changes in smoking behaviors of workers. Results show that while changes in employment type were not associated with the OR of smoking initiation, those whose employment types were changed from temporary to regular employment (OR 0.69; 95% CI, 0.52–0.91) and consecutive temporary employment (OR 0.57; 95% CI, 0.46–0.71) had a decreased likelihood of smoking cessation. Specifically, consecutive temporary employment was most inversely associated with smoking cessation in both men (OR 0.56; 95% CI, 0.44–0.71) and women (OR 0.37; 95% CI, 0.16–0.85).

**Table 4.** Change in employment type and its relation to initiation and cessation of smoking

	Smoking initiation					

	All		Men		Women	

	Cases/*N*	OR (95% CI)	Cases/*N*	OR (95% CI)	Cases/*N*	OR (95% CI)
**Change in employment type** **(year*_t_*_−1_** → **year*_t_*)**						
Regular → Regular	466/9,982	Reference	451/5,705	Reference	15/4,277	Reference
Temporary → Regular	61/2,000	0.96 (0.72–1.29)	54/680	1.01 (0.74–1.38)	7/1,320	1.18 (0.48–2.86)
Regular → Temporary	56/1,576	1.13 (0.84–1.52)	48/595	1.12 (0.81–1.55)	8/981	1.96 (0.86–4.44)
Temporary → Temporary	171/7,857	0.88 (0.71–1.10)	134/2,222	0.87 (0.68–1.11)	37/5,635	1.47 (0.78–2.76)

	Smoking cessation					

	All		Men		Women	

	Cases/*N*	OR (95% CI)	Cases/*N*	OR (95% CI)	Cases/*N*	OR (95% CI)

**Change in employment type** **(year*_t_*_−1_** → **year*_t_*)**						
Regular → Regular	594/4,596	Reference	575/4,545	Reference	19/51	Reference
Temporary → Regular	68/687	0.69 (0.52–0.91)	59/657	0.66 (0.49–0.90)	9/30	0.55 (0.20–1.54)
Regular → Temporary	67/594	0.79 (0.59–1.04)	63/571	0.82 (0.62–1.09)	4/23	0.20 (0.05–0.76)
Temporary → Temporary	216/2,538	0.57 (0.46–0.71)	177/2,352	0.56 (0.44–0.71)	39/186	0.37 (0.16–0.85)

CI, confidence interval; OR, odds ratio.

The models adjusted for gender, age, education, income, marital status, occupation, and survey year.

## DISCUSSION

Our study revealed that temporary employment was significantly associated with an elevated OR of current and high-intensity smoking, as well as a reduced likelihood of smoking cessation. Specifically, workers with shorter duration of employment contract, particularly those with daily employment, exhibited a higher OR of smoking than those with fixed-term or regular employment. Additionally, workers who experienced consecutive periods of temporary employment were less likely to quit smoking in the following year compared to those whose employment position remained regular employment. The association between temporary employment and smoking behaviors is observed both in men and women.

As the descriptive analysis shows, our findings suggest a higher gender gap in the proportion of temporary employment among women than among men, suggesting that women are marginalized into temporary, precarious work in the labor market in South Korea.^23^^,^^24^ Our findings demonstrate that persistent temporary employment can increase the OR of smoking not only in men but also in women. Therefore, given the high prevalence of temporary employment among female workers, policy interventions aimed at enhancing job security can be effective in reducing smoking risk, particularly among female employees.

The findings of our study are consistent with those of previous studies, which revealed that temporary employment is associated with an increased OR of smoking. For instance, previous Korean studies have suggested that temporary employees are at a high likelihood of smoking.^16^^,^^25^ In contrast, a Turkish study found that temporary employment was not associated with current smoking risk.^17^ Due to variations in legal regulations, social protection, and working environments across countries, the association between temporary employment and smoking can manifest differently depending on the cultural context. The major limitation of the above studies is that it has not been discovered whether temporary employment is related to changes in smoking behaviors, as their analyses were based on a cross-sectional design. Therefore, our study contributes to the literature by suggesting that temporary employment may prevent workers from quitting smoking. Additionally, our study shows that workers with prolonged temporary employment had a further reduced likelihood of quitting smoking in both men and women. This finding aligns with those of previous studies that cumulative exposure to precarious employment can increase the risk of addictive behaviors, including smoking and risky alcohol use.^26^^,^^27^ Therefore, our findings suggest that improving job insecurity could be an important factor in promoting healthy lifestyle behaviors among workers.

The mechanism by which temporary employment affects the smoking behavior of workers can be explained by an increased level of job-related stressors. First, workers in temporary employment experience a high level of job insecurity because of their unstable employment positions. Previous studies have shown that job insecurity can increase the risk of adverse health behaviors, such as alcohol consumption and smoking, because temporary employees may engage in addictive behaviors to cope with the stress caused by high job insecurity.^28^^,^^29^ Second, most temporary employees in South Korea are excluded from various social benefits,^30^ which may intensify the impact of job insecurity. In addition, temporary employees lack social support from coworkers,^31^ which facilitates smoking cessation among workers.^32^ Third, previous studies have reported a positive impact of workplace smoking bans on employees’ smoking cessation.^33^^,^^34^ In Korea, a complete ban on indoor smoking has been enforced since 2015, leading some workplaces to implement anti-smoking policies.^35^ Nevertheless, in small-scale businesses and outdoor workplaces with a high prevalence of temporary employees, most of these regulations have remained unenforced.^36^ Given that secondhand smoking exposure and workplace smoking culture can influence employees’ smoking behavior, temporary employees had a reduced likelihood of quitting smoking.

Our study had several limitations. First, it is important to note that our study was based on an observational design; therefore, our findings do not guarantee a causal relationship. The observed associations between temporary employment and smoking behavior might be subject to confounding variables that were not measured in our analysis, limiting our ability to establish causality. Second, multidimensional aspects of job-related stressors that are prevalent in temporary employment, such as job insecurity, high job demands, and lack of job autonomy and social support, were not considered in the analyses because of a lack of information.^37^ Therefore, further studies exploring which job stressors contribute to high smoking risk are warranted to elucidate the exact mechanism of the effect of temporary employment on changes in smoking behavior. Third, as our measurement of smoking status was based on a self-report questionnaire, there might be a possibility of reporting bias.^35^ Therefore, further in-depth research utilizing more objective measurements, such as urine cotinine levels, is needed to verify the conclusions of our study. Fourth, the possibility of reverse causation should be considered. In Korea, smoking has been regarded as a social taboo. Hence, smokers may be at risk of being excluded from regular and stable employment positions compared to non-smokessrs.^38^

Despite these limitations, one strength of our study is its ability to demonstrate that temporary employment is associated not only with current smoking behavior but also with smoking cessation in the subsequent year. Furthermore, our findings exhibit strength in terms of generalizability, as our study sample encompasses workers from various occupational backgrounds.

### Conclusion

Temporary employment is associated with an increased likelihood of current and high-intensity smoking and a decreased likelihood of smoking cessation. Moreover, experiencing temporary employment for consecutive years is inversely associated with smoking cessation in both men and women. Therefore, providing secure employment conditions could be important in reducing smoking prevalence among workers.

## SUPPLEMENTARY MATERIAL

eTable 1. Number of study participants according to survey year and gender

eTable 2. Association between temporary employment and smoking behaviors in men and women

eFigure 1. Proportion of current smokers and temporary employees according to survey year and gender
