# Supplementary material for: Association Between Temporary Employment and Current Smoking and Change in Smoking Behaviors: A Prospective Cohort Study From South Korea (2009–2018)
Source: J Epidemiol. 2024 Oct 5;34(10):459–66. doi: 10.2188/jea.JE20230223 (PMC11405365; doi:10.2188/jea.JE20230223)
Supplement: Supplementary file 1 [file je-34-459-s001.pdf]

eTable 1. Number of study participants according to survey year and gender

Year	All			Men			Women		
	Employment type			Employment type			Employment type		
	Regular employment	Fixed-term	Daily	Regular employment	Fixed-term	Daily	Regular employment	Fixed-term	Daily
2009	2,814 (9.9%)	1,397 (8.6%)	804 (11.0%)	1,938 (10.4%)	576 (8.4%)	414 (11.5%)	876 (9.1%)	821 (8.8%)	390 (10.4%)
2010	2,681 (9.5%)	1,588 (9.8%)	771 (10.5%)	1,883 (10.1%)	683 (9.9%)	382 (10.6%)	798 (8.3%)	905 (9.7%)	389 (10.4%)
2011	2,601 (9.2%)	1,548 (9.5%)	714 (9.7%)	1,789 (9.6%)	648 (9.4%)	350 (9.7%)	812 (8.4%)	900 (9.6%)	364 (9.7%)
2012	2,393 (8.5%)	1,542 (9.5%)	679 (9.3%)	1,612 (8.6%)	672 (9.8%)	342 (9.5%)	781 (8.1%)	870 (9.3%)	337 (9.0%)
2013	2,597 (9.2%)	1,648 (10.1%)	665 (9.1%)	1,746 (9.4%)	728 (10.6%)	334 (9.3%)	851 (8.8%)	920 (9.8%)	331 (8.9%)
2014	2,910 (10.3%)	1,995 (12.3%)	819 (11.2%)	1,958 (10.5%)	864 (12.6%)	393 (10.9%)	952 (9.9%)	1,131 (12.1%)	426 (11.4%)
2015	2,813 (9.9%)	1,844 (11.3%)	743 (10.1%)	1,823 (9.8%)	815 (11.8%)	371 (10.3%)	990 (10.3%)	1,029 (11.0%)	372 (10.0%)
2016	2,845 (10.1%)	1,728 (10.6%)	727 (9.9%)	1,818 (9.7%)	729 (10.6%)	343 (9.5%)	1,027 (10.7%)	999 (10.7%)	384 (10.3%)
2017	3,254 (11.5%)	1,488 (9.2%)	682 (9.3%)	2,020 (10.8%)	589 (8.6%)	325 (9.0%)	1,234 (12.8%)	899 (9.6%)	357 (9.6%)
2018	3,375 (11.9%)	1,473 (9.1%)	729 (9.9%)	2,061 (11.1%)	580 (8.4%)	345 (9.6%)	1,314 (13.6%)	893 (9.5%)	384 (10.3%)

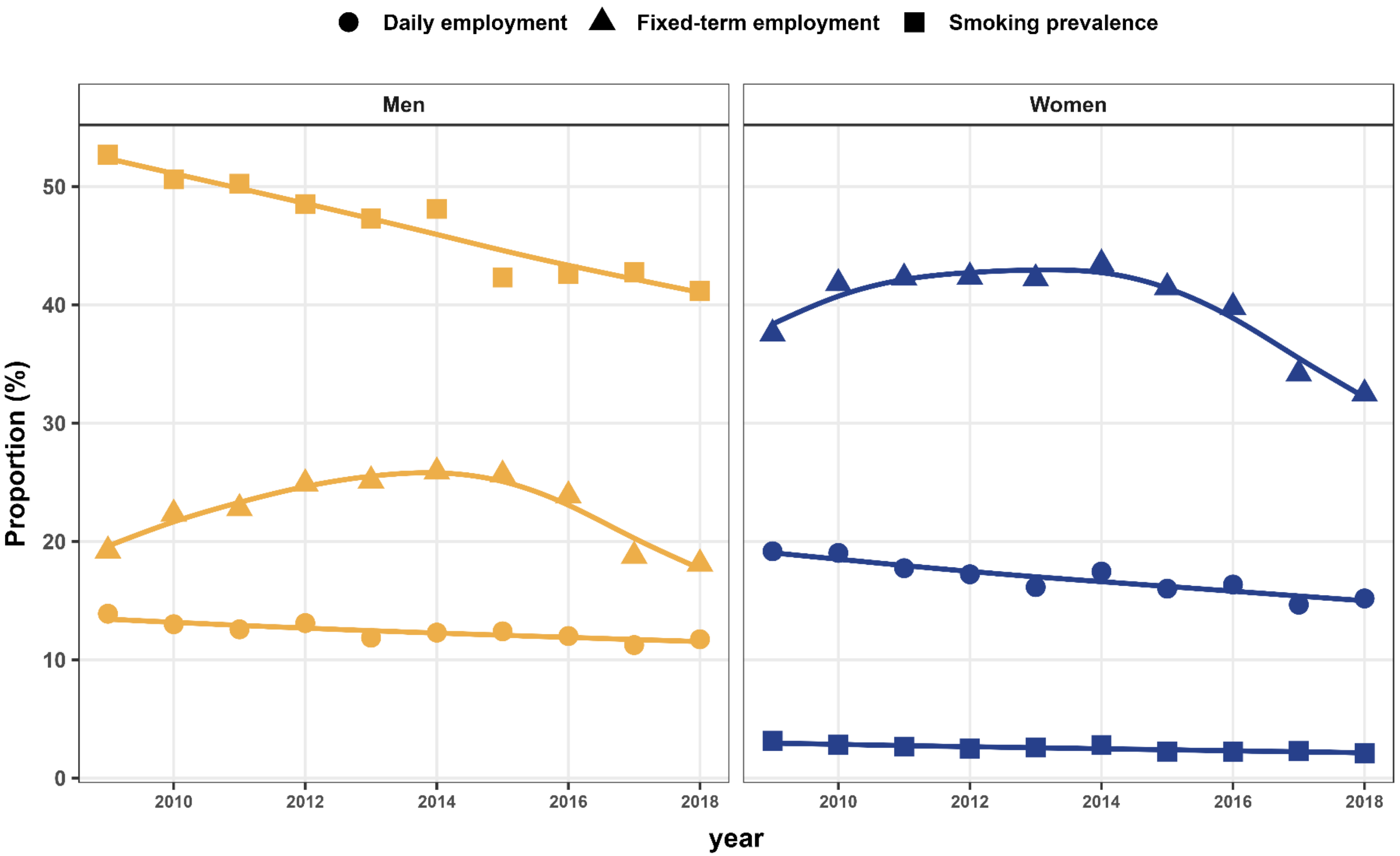

eFigure 1. Proportion of current smokers and temporary employees according to survey year and gender

eTable 2. Association between temporary employment and smoking behaviors in men and women

	Current smoking				High-intensity smoking			
	Men		Women		Men		Women	
	Case/N	OR (95% CI)	Case/N	OR (95% CI)	Case/N	OR (95% CI)	Case/N	OR (95% CI)
**Employment type**								
Regular	8,337/18,648	Reference	144/9,635	Reference	3034/18,648	Reference	12/9,635	Reference
Fixed-term	3,187/6,884	1.13 (1.03–1.25)	253/9,367	1.35 (0.97–1.88)	1454/6,884	1.38 (1.24–1.54)	24/9,367	0.91 (0.38–2.18)
Daily	2,063/3,599	1.74 (1.49–2.04)	173/3,734	1.84 (1.24–2.74)	1212/3,599	2.15 (1.84–2.52)	22/3,734	1.18 (0.52–2.68)

	Smoking initiation				Smoking cessation			
	Men		Women		Men		Women	
	Case/N	OR (95% CI)	Case/N	OR (95% CI)	Case/N	OR (95% CI)	Case/N	OR (95% CI)
**Employment type**								
Regular	647/8,371	Reference	36/7,465	Reference	821/6,849	Reference	33/116	Reference
Fixed-term	210/3,134	0.95 (0.79–1.14)	48/7,670	1.01 (0.63–1.63)	259/2,722	0.77 (0.65–0.91)	58/210	1.05 (0.60–1.83)
Daily	87/1,281	1.06 (0.80–1.41)	21/3,018	0.89 (0.47–1.71)	136/1,770	0.66 (0.52–0.83)	31/153	0.83 (0.43–1.62)

CI, confidence interval; OR, odds ratio.

Model adjusted for gender, age, education, income, marital status, occupation, and survey year.
